# Efficacy and safety of tolperisone *versus* baclofen among Chinese patients with spasticity associated with spinal cord injury: a non-randomized retrospective study

**DOI:** 10.1590/1414-431X2021e11293

**Published:** 2021-09-03

**Authors:** Mingheng Li, Yan Huang, Rongchun Chen, Ning Liu, Shibing Fang

**Affiliations:** 1 Department of Spine Surgery, Ganzhou People's Hospital, Ganzhou, Jiangxi, China; 2 Department of Acupuncture Rehabilitation, Ganzhou Hospital of Traditional Chinese Medicine, Ganzhou, Jiangxi, China

**Keywords:** Baclofen, Barthel Index, Modified Ashworth scale score, Modified Medical Research Council score, Spasticity, Tolperisone

## Abstract

There are many medications available to treat spasticity, but the tolerability of medications is the main issue for choosing the best treatment. The objectives of this study were to compare the efficacy and adverse effects of tolperisone compared to baclofen among patients with spasticity associated with spinal cord injury. Patients received baclofen plus physical therapy (BAF+PT, n=135) or tolperisone plus physical therapy (TOL+PT, n=116), or physical therapy alone (PT, n=180). The modified Ashworth scale score, the modified Medical Research Council score, the Barthel Index score, and the Disability Assessment scale score were improved (P<0.05 for all) in all the patients at the end of 6 weeks compared to before interventions. After 6 weeks, the overall coefficient of efficacy of the intervention(s) in the BAF+PT, TOL+PT, and PT groups were 1.15, 0.45, and 0.05, respectively. The patients of the BAF+PT group reported asthenia, drowsiness, and sleepiness and those of the TOL+PT group reported dyspepsia and epigastric pain as adverse effects. When comparing drug interventions to physical therapy alone, both baclofen plus physical therapy and tolperisone plus physical therapy played a significant role in the improvement of daily activities of patients. Nonetheless, baclofen plus physical therapy was tentatively effective. Tolperisone plus physical therapy was slightly effective. In addition, baclofen caused adverse effects related to the sedative manifestation (Level of Evidence: III; Technical Efficacy Stage: 4).

## Introduction

Motor neuron dysfunction due to defects in inhibitory descending motor pathways of the spinal cord leads to spasticity (1,2). Hyperexcitability of the stretch reflex, exaggerated tendon jerks, and a velocity-dependent increase in tonic stretch reflexes are clinical signs of spasticity (1). Another characteristic is an increase in muscle tone (3).

Treatment of spasticity is based on the rehabilitation of patients to improve daily activities (2). Muscle relaxants work through polysynaptic reflex mechanisms. Therefore, they are good for the treatment of spasticity associated with spinal cord injury (4). Baclofen is a γ-aminobutyric acid β-agonist, a central muscle relaxant, and approved by the United States Food and Drug Administration (USFDA) for treatment of spasticity associated with spinal cord injury (5). It is effective within a week of intervention (1), but it is an addictive drug and causes sedation, dizziness, drowsiness, and other adverse effects during treatment (2). Tolperisone is also a centrally acting muscle relaxant (sodium and calcium channel blocker at brain stem) and has no sedation and withdrawal symptoms (1). A prospective study in the Indian population (1) shows the superiority of tolperisone over baclofen among patients with spasticity associated with spinal cord injury, cerebral palsy, or post-stroke, but this prospective study had a small sample size.

For the pharmacological management of spasticity, baclofen, dantrolene, tizanidine, and diazepam are commonly used initial medications (6,7). Baclofen is generally considered the first-line treatment in spinal cord injury and can be very effective despite its side effects (3). On the other hand, tolperisone is mainly prescribed for acute muscle spasms and is not approved by the USFDA for the treatment of spasticity. The comparison of efficacy and safety of these two drugs has not been studied in depth.

The objectives of this non-randomized retrospective analysis were to compare muscle tone, muscle strength, functional outcomes, disability assessment, and treatment-emergent adverse effects of tolperisone plus physical therapy with those of baclofen plus physical therapy and non-treatment interventions among Chinese patients with spasticity associated with spinal cord injury.

## Material and Methods

### Ethical consideration and consent to participate

The designed protocol (GPH/CL/04/20 dated 5 August 2020) was approved by the Ganzhou People's Hospital review board. The study adhered to the law of China and the 2008 Declaration of Helsinki. An informed consent form was signed by patients and/or relatives (the legally authorized person) of the patients regarding intervention(s) and publication of the anonymized information of patients in the form of an article before treatment.

### Inclusion and exclusion criteria

Patients aged 18 years and above, experiencing spasticity of hip adductor muscles, medial hamstring muscle, or the lower limbs associated with spinal cord injuries (6 months of history), and requiring rehabilitation in performing daily activities (patients had modified Ashworth scale 2 or less, modified Medical Research Council score 2 or less, Barthel Index functional outcomes score 50 or less before treatments) were included in the analysis.

Patients aged below 18 years, who had an orthopedic fracture, concomitant neurological disease before treatment, were pregnant, and who had a loss of locomotion other than spasticity were excluded from the analysis.

### Sample size calculation

The study was based on the assumption that 80±5% of patients would reach a modified Ashworth score of more than 2 and a 15% drop‐out over the intervention period of treatment and/or non-treatment intervention(s). The sample size was calculated on this assumption of muscle tone, a 5% two-sided type-I error (α=0.05), and 80% power (β=0.2) at 95% level of confidence. The sample size (minimum patients required in each group) was 115 (8).

### Patient groups and therapy

A total of 135 patients with spasticity who had been using dextromethorphan for cough, viral infections, and/or myasthenia gravis received 5 mg baclofen (BAF; Actavis-UK, Ltd., UK) three times a day. The dose was increased by 5 mg/week. The titration was carried out up to 80 mg/day (2). Patients also received physical therapy. These patients were included in the BAF+PT group. A total of 116 patients with spasticity who had lactose intolerance and/or stomach ulcer, problems with lungs, bladder, and/or diabetes mellitus received 150 mg/day in three divided doses of tolperisone (TOL; Myolax, Incepta Pharmaceuticals Ltd., Bangladesh). Titration was carried out up to 600 mg/day (1). Patients also received physical therapy. These patients were included in the TOL+PT group. A total of 180 patients with spasticity who had impairment of kidney function, hepatic function, and heart functions, were on antidepressant therapy, on Alzheimer's disease therapy, were planned for surgery under anesthesia, had a history of skin allergies, porphyria (an inherited condition causing skin blisters, abdominal pain, and agitation), and/or epilepsy (susceptible to baclofen and tolperisone) received physical therapy only (9,10). These patients were included in the PT group. The adjustment of baclofen and tolperisone dose was made at 2, 4, and 6 weeks.

### Physical therapy

Physical therapy included 1 h/day locomotor training, i.e., body weight-supported treadmill training, walking practice on the ground or on a treadmill, stepping practice, and walking practice in and out of exercise stations (11). Intensive task-specific training, for example, walking, sit-to-stand transfers, and standing, was also included (12). Physical therapy was performed by physiotherapists with a minimum of 3 years of experience at institutes. Physiotherapists were blinded for the groups. Physiotherapy was different according to the lesion (cervical, dorsal, or lumbar).

### Outcome measures

Outcome measures were evaluated by physiotherapists with a minimum of 3 years of experience at institutes at the end of 2, 4, and 6 weeks of treatment and/or non-drug intervention.

The tone of a spastic muscle was evaluated using the modified Ashworth scale and the strength of a spastic muscle was evaluated using the modified Medical Research Council score (2), as shown in Table 1.

**Table 1:** Grading of the modified Ashworth scale and the modified Medical Research Council score.

Grade	Situation
Modified Ashworth scale	
0	No significant improvement
1	Slight improvement
2	Significant improvement but affected part is not moved easily
3	Significant improvement and affected part is moved easily
4	Muscle tone is increased but the passive movement is difficult
5	Muscle tone is increased and passive movement is reported
Modified Medical Research Council score	
0	No contraction
1	Small contraction
2	Movement with gravity
3	Movement reported against gravity but not against resistance
4	Movement reported against gravity and slight resistance
5	Movement reported against gravity and strong resistance
6	Normal power

Functional outcomes were evaluated using the Barthel Index score for 10 activities. The activities included use of the toilet, bladder continence, bowel continence, ambulation, feeding, bathing, dressing, grooming, stair climbing, and transfers. Each activity has a score range of 0 to 10: 0 indicates dependency and 10 indicates independency (perform activity without the help of a human). The total score is 100 (13).

The Disability Assessment scale score was evaluated as 0: no disability (full activity); 1: slight disability; 2: moderate disability; 3: severe disability (limited activity); and 4: extreme disability (no activity) (14).

The coefficient of efficacy after 6 weeks of intervention for each outcome measure was evaluated as per Equations 1, 2, 3, and 4, respectively:

$$\text{Coefficient of the efficacy of muscle tone after 6 weeks of treatment =}\frac{\text{Number of patients with 4 or 5 modified Ashworth scale after 6 weeks of intervention}}{\text{Number of patients with 0, 1, or 2 modified Ashworth scale after 6 weeks of intervention}}$$ Equation 1

$$\text{Coefficient of the efficacy of muscle strength after 6 weeks of treatment =}\frac{\text{Number of patients with 5 or 6 modified Medical Research Council score after 6 weeks of intervention}}{\text{Number of patients with 0, 1, or 2 modified Medical Research Council score after 6 weeks of intervention}}$$ Equation 2

$$\text{Coefficient of the efficacy of functional outcomes after 6 weeks of treatment =}\frac{\text{Number of patients with}\geq \text{75 Barthel Index score after 6 weeks of intervention}}{\text{Number of patients with <50 Barthel Index score after 6 weeks of intervention}}$$ Equation 3

$$\text{Coefficient of the efficacy of Disability Assessment scale score after 6 weeks of treatment =}\frac{\text{Number of patients with 0 or 1 Disability Assessment scale score after 6 weeks of intervention}}{\text{Number of patients with 3 or 4 Disability Assessment scale score after 6 weeks of intervention}}$$ Equation 4

The overall coefficient of efficacy after 6 weeks of intervention was the sum of the coefficient of the efficacy of each outcome measure after 6 weeks of interventions divided by the number of outcomes evaluated (i.e., 4) as per Equation 5, where n=number of outcome measures. If the overall coefficient of efficacy was ≥3, then treatment was considered highly effective, from 2-2.99, sufficiently effective, from 1-1.99, tentatively effective, from 1-0.40, slightly effective, and <0.40, then treatment was considered ineffective (1).

$$\text{Overall coefficient of efficacy =}\frac{\sum_{1}^{\text{n}}\text{Coefficient of efficacy}}{\text{n}}$$ Equation 5

Data regarding treatment-emergent adverse effects of patients during 6 weeks of treatment and/or non-drug interventions were retrospectively collected from the patients' records at the institutes.

### Statistical analysis

InStat, 3.01 (GraphPad, USA) was used for statistical analysis. One-way analysis of variance (ANOVA) followed by the Tukey test (considering critical value (q)>3.322 as significant) for continuous and ordinal variables between groups and the repeated measures ANOVA followed by the Tukey test (considering critical value (q)>3.646 as significant) for continuous and ordinal variables within the group were performed for statistical analysis (2). The Fisher exact test (for two columns and two rows) or the chi-squared of independent samples (more than two columns and two rows) was performed for categorical data. Results were significant if P<0.05.

## Results

### Study population

**Figure 1:**
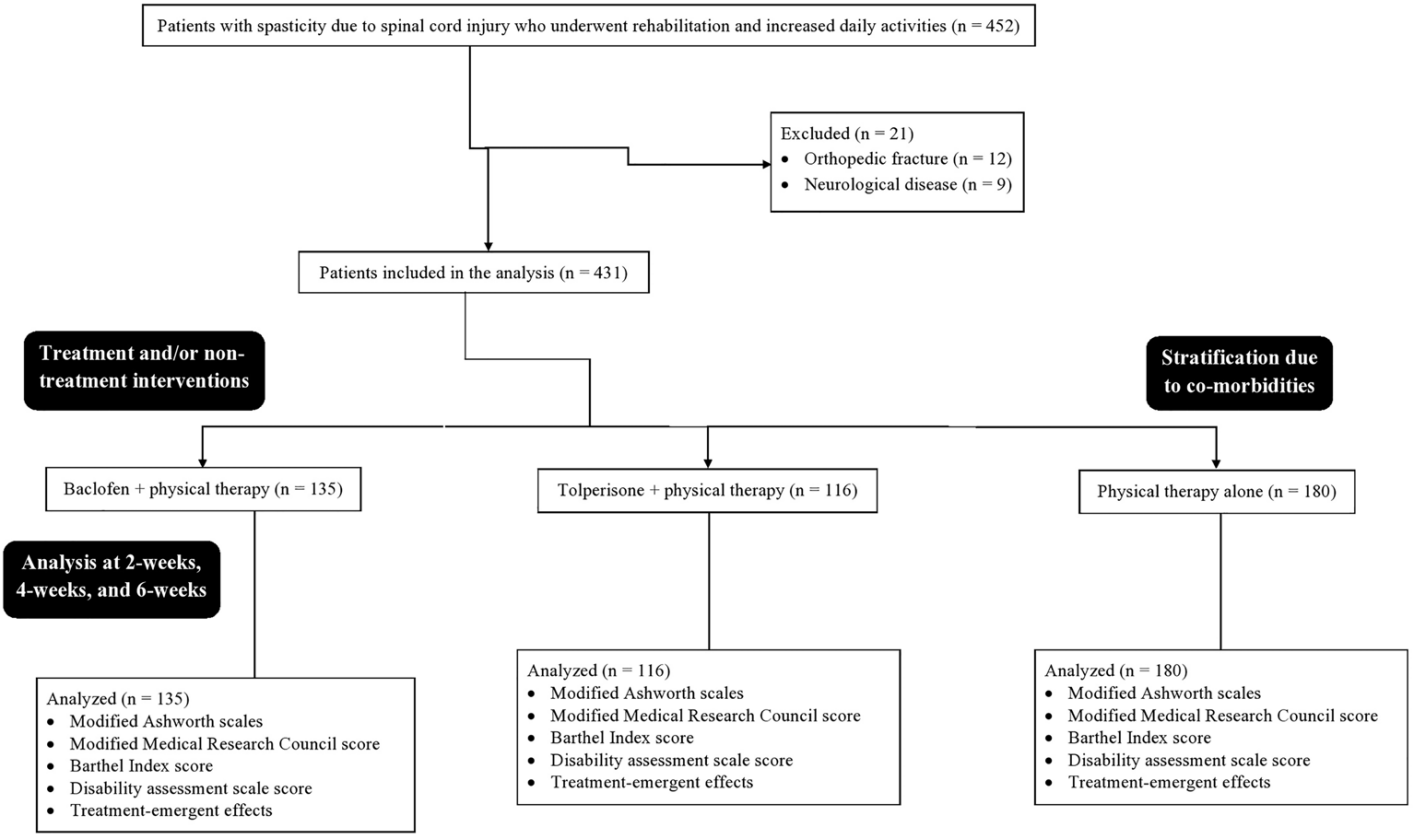
The flow diagram of management of spasticity.

From January 15, 2018 to July 1, 2020, a total of 452 patients were reported with spasticity at the Department of Spine Surgery of the Ganzhou People's Hospital, China, and Ganzhou Hospital of Traditional Chinese Medicine, China. Among them, 12 had an orthopedic fracture and 9 had neurological disease(s). Therefore, data of these patients (n=21) were excluded from the analysis. Data of treatment efficacy and scores of the scales of 431 patients were retrospectively collected after obtaining written approval from Institutions. The flow diagram of management of spasticity is shown in Figure 1.

### Demographic and spasticity characteristics

At baseline, there were no significant differences among the groups for the demographic and spasticity characteristics (P>0.05 for parameters, Table 2). Patients who had other major disorders, like myasthenia gravis and diabetes mellitus, were taking other medicines together with the study interventions.

**Table 2:** Demographic and spasticity characteristics before interventions.

Characteristics	BAF+PT (n=135)	TOL+PT (n=116)	PT (n=180)	P-value
Age (years)	37.61±5.15	38.12±3.15	39.01±6.16	0.053
Gender				
Male	64 (47)	61 (53)	79 (44)	0.343
Female	71 (53)	55 (47)	101 (56)	
Body weight (kg)	57.12±7.15	56.41±6.49	57.81±8.15	0.282
Height (cm)	153.52±7.82	151.49±8.15	152.41±8.55	0.147
Body mass index (kg/m^2^)	24.28±1.34	24.59±1.35	24.71±1.89	0.058
History of spasticity (days)	210±22	215±19	212±21	0.164
Spasticity side	92 (68)	71 (61)	109 (61)	0.340
Dominant side	43 (32)	45 (39)	71 (39)	
Non-dominant side				
Spasticity associated with spinal cord injury				
Hip adductors muscle	32 (24)	24 (21)	32 (18)	0.558
Medial hamstring muscle	31 (23)	21 (18)	41 (23)	
Lower limbs	72 (53)	71 (61)	107 (59)	
Time of spinal injury (days)	236±17	231±31	238±29	0.083
Comorbidities				
History of viral infections	15 (11)	0 (0)	0 (0)	<0.0001
History of myasthenia gravis	5 (4)	0 (0)	0 (0)	
Lactose intolerance	0 (0)	35 (30)	0 (0)	
Stomach ulcer	0 (0)	7 (6)	0 (0)	
Diabetes mellitus	0 (0)	35 (30)	0 (0)	
Epileptic	0 (0)	0 (0)	11 (6)	
Kidney function impairments	0 (0)	0 (0)	21 (12)	
Hepatic function impairments	0 (0)	0 (0)	18 (10)	
On antidepressant therapy	0 (0)	0 (0)	22 (12)	

Continuous and ordinal variables are reported as means±SD. Categorical variables are reported as frequency (percentages). One-way ANOVA (for continuous variables) and the chi-squared of independent samples test (for categorical variables) were used for statistical analyses. P*<*0.05 was considered significant. Groups: BAF+PT: baclofen plus physical therapy; TOL+PT: tolperisone plus physical therapy; PT: physical therapy.

### Outcome measures

#### Muscle tone

**Figure 2:**
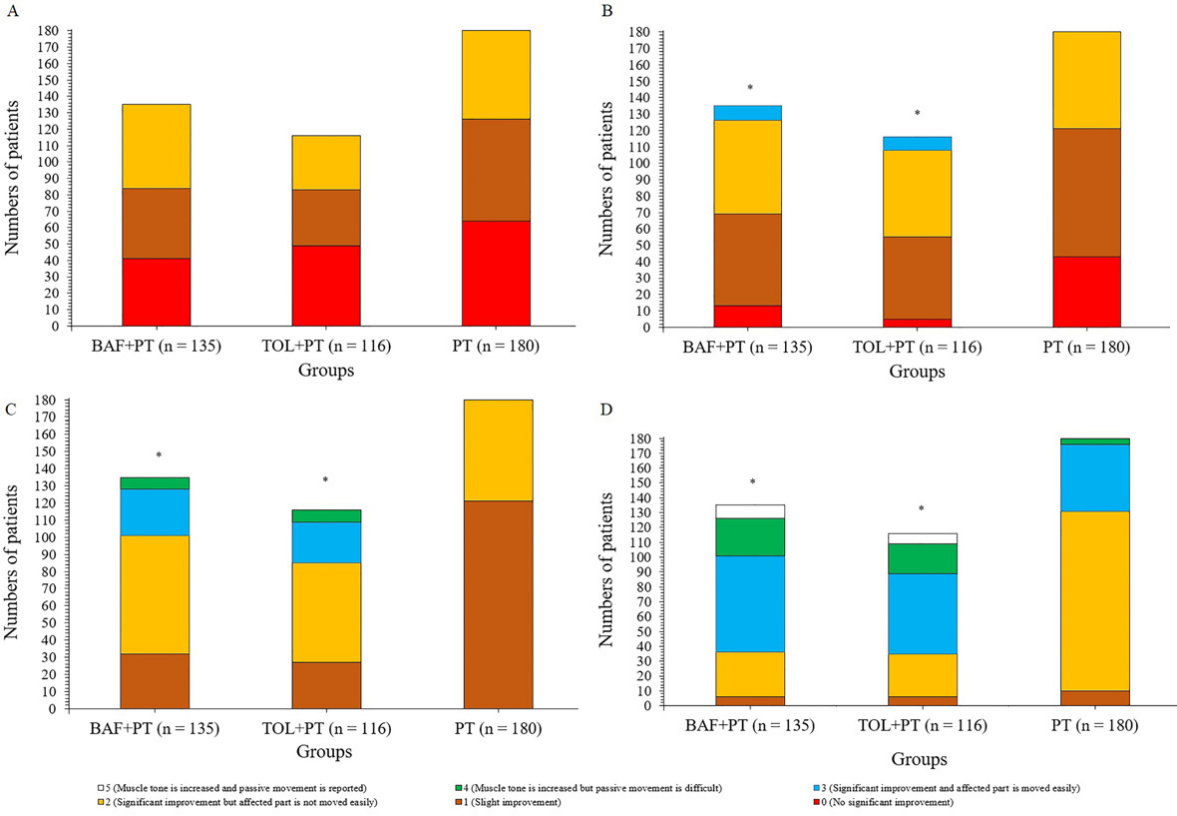
The modified Ashworth scales score of patients at different points of evaluation. **A**, Before the start of interventions. **B**, **C**, and **D**, at 2, 4, and 6 weeks after the start of interventions, respectively. *P<0.05 compared to the PT group (ANOVA). BAF+PT: baclofen plus physical therapy; TOL+PT: tolperisone plus physical therapy; PT: physical therapy alone.

At baseline, there were no significant differences for the modified Ashworth scale score among groups (Figure 2A, P=0.122). At 2, 4, and 6 weeks after the start of interventions (Figure 2B-D), patients of the BAF+PT and TOL+PT groups had improved the modified Ashworth scale score compared to those of the PT group (P<0.05 and q>3.322 for all). Within one week, the BAF+PT (P<0.0001, q=10.959), TOL+PT (P<0.0001, q=17.279), and PT (P<0.0001, q=4.841) groups had a significant improvement of muscle tone. At all time-points, the three interventions showed an improvement of the modified Ashworth scale score of patients compared to baseline (P<0.05 and q>3.646 for all). The detailed results of the modified Ashworth scale score analysis are reported in Supplementary Table S1.

#### Muscle strength

**Figure 3:**
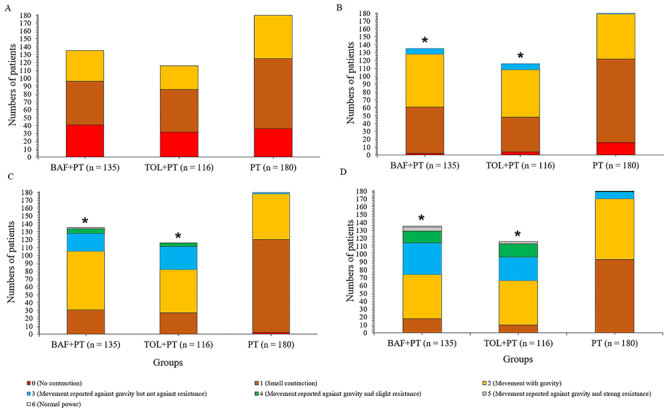
The modified Medical Research Council score of patients at different points of evaluation. **A**, Before the start of interventions. **B**, **C**, and **D**, at 2, 4, and 6 weeks after the start of interventions, respectively. *P<0.05 compared to the PT group (ANOVA). BAF+PT: baclofen plus physical therapy; TOL+PT: tolperisone plus physical therapy; PT: physical therapy alone.

At baseline, there was no significant difference for the modified Medical Research Council score of patients among groups (P=0.182; Figure 3A). At 2 (Figure 3B), 4 (Figure 3C), and 6 (Figure 3D) weeks, patients of BAF+PT and TOL+PT groups had improved scores compared to those of the PT group (P<0.05 and q>3.322 for all). Within one week, the BAF+PT (P<0.0001, q=13.016), TOL+PT (P<0.0001, q=13.529), and PT (P<0.0001, q=5.369) groups had significantly better muscle strength compared to baseline (P<0.05 and q>3.646 for all) (Supplementary Table S2).

#### Functional outcomes

**Figure 4:**
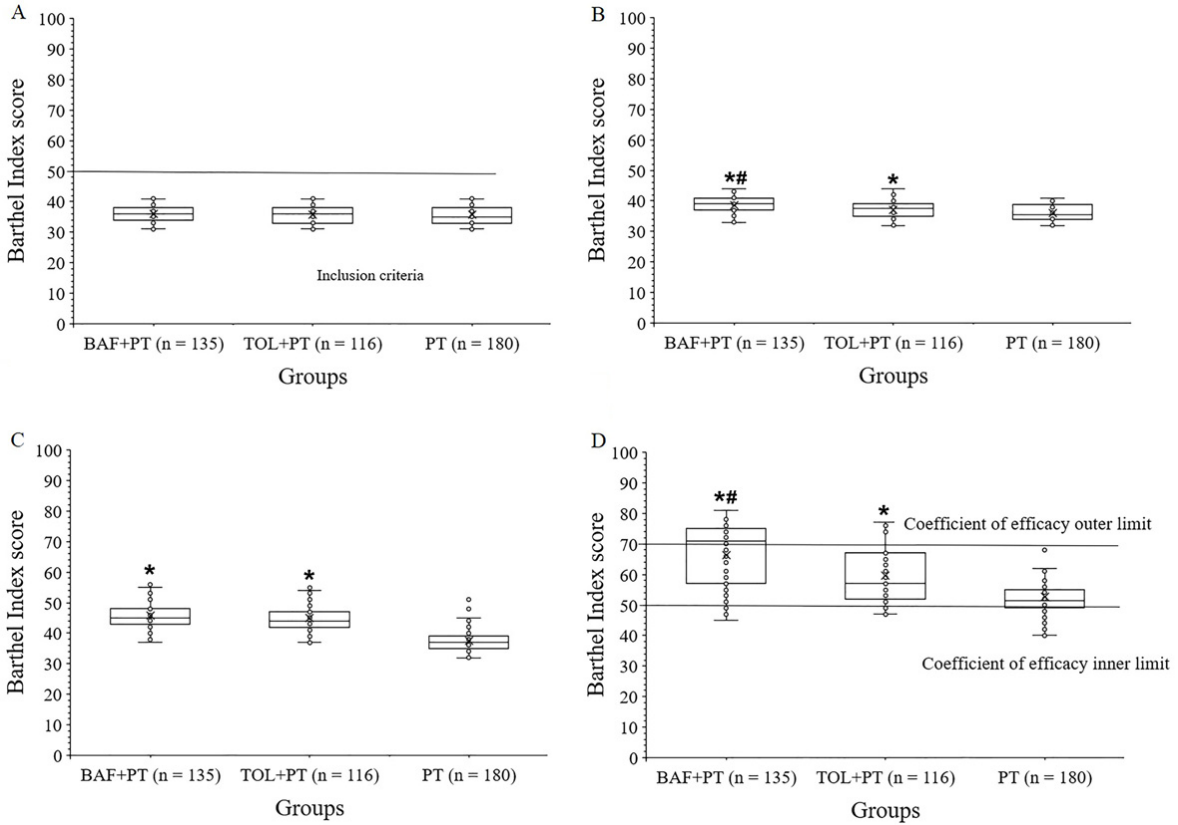
The Barthel Index score at different points of evaluation. **A**, Before the start of interventions. **B**, **C**, and **D**, at 2, 4, and 6 weeks after the start of interventions, respectively. Data are reported as medians and interquartile range. *P<0.05 compared to the PT group; ^#^P<0.05 compared to TOL+PT group (ANOVA). BAF+PT: baclofen plus physical therapy; TOL+PT: tolperisone plus physical therapy; PT: physical therapy alone. A score of 0 indicates patient dependency and ≥95 indicates patient independency.

 At baseline there was no significant difference for the Barthel Index score among groups (Figure 4A; P=0.606). At 2 weeks, patients of the BAF+PT group had improved scores compared to the TOL+PT and PT groups (Figure 4B). At four weeks, patients of the BAF+PT and TOL+PT groups had improved scores compared to the PT group (Figure 4C). At six weeks, patients of the BAF+PT group had improved scores compared to the TOL+PT and PT groups (Figure 4D). Within one week, only the BAF+PT group (P<0.0001, q=6.138) had a significant improvement of the Barthel Index score. The details of the Barthel Index score analyses are reported in Supplementary Table S3.

#### Disability Assessment scale score

**Figure 5:**
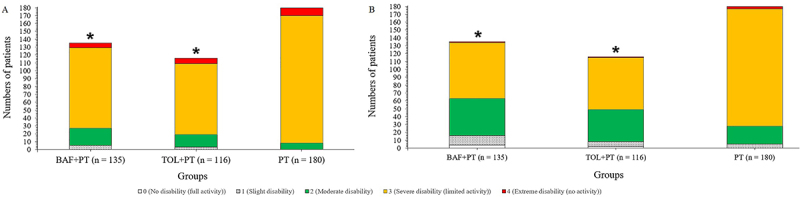
The Disability Assessment scale score of patients at different points of evaluation. **A** and **B**, 4 and 6 weeks after the start of interventions, respectively. *P<0.05 compared to the PT group (ANOVA). BAF+PT: baclofen plus physical therapy; TOL+PT: tolperisone plus physical therapy; PT: physical therapy alone.

At baseline and two weeks, there was no significant difference for the Disability Assessment scale score of patients among groups. At four (Figure 5A) and six weeks (Figure 5B), patients of the BAF+PT and TOL+PT groups had an improved score compared to the PT group (P<0.05 and q>3.322 for both). At all time-points, there was an improvement of the Disability Assessment scale score in patients of the BAF+PT, TOL+PT, and PT groups compared to baseline (P<0.05 and q>3.646 for all) (Supplementary Table S4).

### Coefficient of efficacy

A total of 6 weeks after the start of the interventions, the interventions of BAF+PT were tentatively effective, the interventions of TOL+PT were slightly effective, and only PT was ineffective (Table 3).

**Table 3:** Coefficient of efficacy 6 weeks after the start of interventions.

Outcome measures	Coefficient of efficacy		
	BAF+PT	TOL+PT	PT
Number of patients	135	116	180
Muscle tone	0.89	0.75	0.03
Muscle strength	0.08	0.05	0.00
Functional outcomes	3.42	0.88	0.13
Disability Assessment scale score	0.22	0.12	0.03
Overall (sum of coefficient of the efficacy of all evaluated outcomes/numbers of outcomes evaluated)	1.15	0.45	0.05

Groups: BAF+PT: baclofen plus physical therapy; TOL+PT: tolperisone plus physical therapy; PT: physical therapy.

### Treatment-emergent adverse effects

During the 6 weeks of interventions, the BAF+PT group experienced asthenia, drowsiness, hypoesthesia, paresthesia, sweating, sciatica, vertigo, sleepiness, nausea, amenorrhea, and anorexia as adverse effects. Patients of the TOL+PT group experienced dyspepsia and epigastric pain as adverse effects (Table 4).

**Table 4:** Adverse effects during 6 weeks of interventions.

Adverse effects	BAF+PT (n=135)	TOL+PT (n=116)	PT (n=180)	P-value	Comparisons (q-value)		
					BAF+PT *vs* TOL+PT	BAF+PT *vs* PT	TOL+PT *vs* PT
Headache	2 (1)	2 (2)	3 (2)	0.987	N/A	N/A	N/A
Asthenia*	39 (29)	5 (4)	2 (1)	<0.0001	9.671	12.153	1.339
Hyposthenia*	9 (7)	1 (1)	2 (1)	0.004	3.978	4.234	0.182
Cramps	5 (4)	2 (2)	2 (1)	0.269	N/A	N/A	N/A
Paresthesia*	3 (2)	0 (0)	0 (0)	0.036	2.998	3.334	N/A
Sweating*	3 (2)	0 (0)	0 (0)	0.036	2.998	3.334	N/A
Sciatica*	4 (3)	0 (0)	0 (0)	0.012	3.476	3.865	N/A
Vertigo*	3 (2)	0 (0)	0 (0)	0.036	2.998	3.334	N/A
Sleepiness*	13 (10)	0 (0)	1 (1)	<0.0001	6.264	6.532	0.382
Nausea*	5 (4)	0 (0)	0 (0)	0.004	3.901	4.337	N/A
Musculoskeletal stiffness	2 (1)	0 (0)	5 (3)	0.181	N/A	N/A	N/A
Amenorrhea*	3 (2)	0 (0)	0 (0)	0.036	2.998	3.334	N/A
Anorexia*	3 (2)	0 (0)	0 (0)	0.036	2.998	3.334	N/A
Dyspepsia^#^	0 (0)	7 (6)	1 (1)	0.0004	5.068	0.519	4.893
Epigastric pain^#^	0 (0)	8 (7)	1 (1)	0.0001	5.485	0.491	5.363
Hypochondrial pain	1 (1)	0 (0)	0 (0)	0.335	N/A	N/A	N/A
Hypotonia	0 (0)	1 (1)	0 (0)	0.258	N/A	N/A	N/A
Insomnia	0 (0)	2 (2)	1 (1)	0.251	N/A	N/A	N/A
Itching	0 (0)	1 (1)	0 (0)	0.258	N/A	N/A	N/A
Sciatica	1 (1)	0 (0)	0 (0)	0.335	N/A	N/A	N/A
Vertigo	2 (2)	1 (1)	0 (0)	0.286	N/A	N/A	N/A
Drowsiness*	25 (19)	0 (0)	0 (0)	<0.0001	9.482	10.544	N/A

One-way ANOVA was used for statistical analysis. The Tukey test was used for *post hoc* analysis. A P<0.05 and q>3.322 were considered significant. N/A: Not applicable. *Significant baclofen-emergent adverse effects. ^#^Significant tolperisone-emergent adverse effects. BAF+PT: baclofen plus physical therapy; TOL+PT: tolperisone plus physical therapy; PT: physical therapy alone.

## Discussion

The study found that baclofen plus physical therapy and tolperisone plus physical therapy successfully improved the scales’ scores at the end of the 6-week interventions compared to physical therapy alone. The results are in agreement with those of a trial performed in an Indian population (1). The Indian trial (1) was with children and adults. However, the current study was with adults (age >18 years) only. Also, the Indian trial (1) administered up to 80 mg/day baclofen to children while the maximum daily dose should not be more than 60 mg/day for children >8 years of age (9).

Baclofen is a *γ*-aminobutyric acid β-agonist and is successful in the restoration of the movement and strength of paralyzed muscles because it acts on the central nervous system (15). The membrane-stabilizing action of tolperisone has improved outcome measures of patients suffering from spasticity (16). The physical therapy program that the current study described was inappropriate as it should have included stretching exercises or active range of motion exercises, treatment modalities such as heat/cold therapy, transcutaneous electrical nerve stimulation, electrical stimulation, or functional electrical stimulation (17), which are the other techniques that can be used for the non-pharmacological management of spasticity (1 [Bibr B02] 3). Physical therapy provides sensory input from the periphery and motor input from the sensorimotor cortex onto the damaged spinal cord, which is not enough for structural re-organization in spasticity (12). The use of other physical therapies that could be useful in spastic patients, for instance, high intensity noisy mechanical stimulation to reduce the monosynaptic reflex (18) and Chinese therapies including acupuncture or electroacupuncture (19) should be considered to improve motor functions of patients with spasticity due to spinal cord injuries.

The study found that treatments were tentatively effective in the BAF+PT group and were slightly effective in the TOL+PT group. The results of the coefficient of efficacy of the current study did not agree with those of previous trials (1,20). The reason for the contradictory results was that one trial (1) was performed with a small sample size and the other trial (20) had fewer outcome measures, which can lead to type-I error.

The results of adverse effects of the current study agreed with those of previous trials (1,2,15). Tolperisone directly affects the spinal cord and has imitating effects on the neurotransmitters because it inhibits the synaptic influx of calcium ions, which is the efflux of the neurotransmitters. Because baclofen has a *γ*-aminobutyric acid β-agonist action on the brain (1), it has more treatment-related adverse effects than tolperisone. Tolperisone has no adverse effects related to sedative manifestation. Oral baclofen has poor acceptance by patients because of adverse effects (15). Concerning treatment-emergent adverse effects, tolperisone plus physical therapy is the choice of treatment for the management of spasticity due to spinal cord injuries. The treatments appeared comparable, with an adverse event profile that favored tolperisone. Tolperisone is recommended to patients who are susceptible to adverse effects related to sedative manifestation.

There are several limitations of the study that have to be reported. For example, combined effects of baclofen plus tolperisone plus physical therapy were not evaluated. The follow-up time was of 6 weeks only. The study was a non-randomized retrospective analysis. There was no information available about critical points, such as the lesion's level, the lesion's timing, and type of treatment before the study started. A prospective well-designed study is recommended. An injury in the cervical spine is very different from an injury in the dorsal spine or lumbar spine and, of course, the treatment and the physiological responses to medication are different. The type of injuries (e.g., traumatic or non-traumatic) was not discussed. Retrospective studies are observational, non-randomized studies that are subject to selection bias with no control over confounding variables, which can cause an overestimation or underestimation of the association between specific interventions and treatment effects. The study did not report the number of patients who discontinued the treatment due to a lack of therapeutic effects or side effects related to medication. These variables are highly relevant in studies evaluating the efficacy and safety of drug treatments or interventions. The reported side effects are not systematically recorded in clinical practice (for baclofen/tolperisone and physiotherapy). Therefore, the reported prevalence of side effects and the derived conclusions about the safety of the compared treatments lack validity. The study only included paraplegic patients.

### Conclusions

Baclofen plus physical therapy as well as tolperisone plus physical therapy had a significant role in the improvement of daily activities of patients with spasticity due to spinal cord injuries. However, baclofen plus physical therapy was tentatively effective and tolperisone plus physical therapy was slightly effective. Baclofen had important adverse effects related to sedative manifestation.

## Supplementary Material

Click to view [pdf].
